# Parent and child interactions with two contrasting anti-obesity advertising campaigns: a qualitative analysis

**DOI:** 10.1186/1471-2458-14-151

**Published:** 2014-02-11

**Authors:** Samantha L Thomas, Timothy Olds, Simone Pettigrew, Heather Yeatman, Jim Hyde, Christine Dragovic

**Affiliations:** 1 School of Health and Society, Faculty of Social Sciences, Building 234 (iC Enterprise1), Innovation Campus, University of Wollongong, Northfields Avenue, Wollongong, NSW 2522, Australia; 2 Australian Health Services Research Institute, University of Wollongong, Northfields Avenue, Wollongong, NSW 2522, Australia; 3 Health and Use of Time (HUT) Group, University of South Australia, Adelaide, Australia; 4 School of Psychology and Speech Pathology, Curtin University, Perth, Australia; 5 Deakin Population Health SRC, Deakin University, Deakin, Australia

**Keywords:** Mass media campaigns, Parents, Children, Obesity

## Abstract

**Background:**

Social marketing has been proposed as a framework that may be effectively used to encourage behaviour change relating to obesity. Social advertising (or mass media campaigning) is the most commonly used social marketing strategy to address the issue of obesity. While social advertising has the potential to effectively communicate information about obesity, some argue that the current framing and delivery of these campaigns are ineffective, and may cause more harm than good.

**Methods:**

We used a qualitative advertising reception study. 150 family groups (comprised of 159 parents and 184 children) were shown two Australian government anti-obesity advertisements: *Measure Up* (focused on problems associated with obesity) and *Swap It* (focused on solutions for obesity). Families were engaged in a discussion about the visual appeals, verbal messages and their perceptions about the impact of the advertisements on behavioural change. Open coding techniques and a constant comparative method of analysis was used to interpret the data.

**Results:**

Many parents had strong personal resonance with the visual imagery within the campaigns. While *Swap It* had strong ‘likeability’ with children, many children believed that the messages about overweight and obesity were less personally relevant because they did not perceive themselves to be overweight. The content and delivery style of the verbal messages (the serious risk focused message in *Measure Up* compared to the upbeat, fun practical message in *Swap It*) influenced how different audiences (parents and children) interpreted the information that was presented. Parents assimilated practical and instructive messages, while children assimilated messages about weight loss and weight gain. Parents and children recognised that the campaigns were asking individuals to take personal responsibility for their weight status, and were at times critical that the campaigns did not tackle the broader issues associated with the causes and consequences of obesity. The lack of practical tools to encourage behavioural change was a key barrier for obese parents.

**Conclusions:**

Well-funded, targeted social marketing campaigns will play an important role in the prevention and management of obesity. It is important that these campaigns are comprehensively evaluated and are backed up with structural supports to enable and encourage population subgroups to act upon messages.

## Background

Obesity is recognised as one of the world’s most pressing public health issues, with many nations experiencing marked increases in the prevalence of obesity in recent decades [1]. In Australia, about 25% of adults are obese, and an additional 37% are overweight [2], and 20-25% of children and adolescents overweight or obese [3]. While recent evidence suggests that overweight and obesity in children may have plateaued in Australia and many other countries [4,5], prevalence remains high. As a result, efforts to prevent and reduce obesity continue to be a public health imperative.

A key challenge for public health and health promotion practitioners is how to effectively engage individuals in behaviours which prevent, manage or reduce obesity. Simplistic ‘eat less, exercise more’ messages that overwhelmingly focus on individual responsibility appear to have had limited impact on changing behaviour. Furthermore, public health messages warning individuals of the risks associated with obesity sit alongside industry marketing campaigns which actively and seductively engage individuals in obesity promoting consumption and activity practices. Nevertheless, social marketing is an important framework which, if effectively applied and sustained within a comprehensive public health approach to obesity prevention, may stimulate changes in obesity-related food consumption and physical activity behaviours [6,7]. Using a range of promotional techniques, including social advertising (or mass media campaigning), social marketing programs apply the principles used in commercial marketing for social good (of individuals or society) rather than commercial goals [8-11]. While individual behaviour change is at the heart of any social marketing initiative, a comprehensive social marketing approach recognises the importance of the structural environment in shaping health and social behaviours, and advocates action at the downstream, midstream and upstream levels [12].

Despite the broad base of social marketing, social advertising is the most visible and widely used part of the social marketing mix [13-18]. Social advertising aims to inform individuals of the risks and consequences of engaging in a particular detrimental behaviour and to stimulate positive behavioural change. Notwithstanding its widespread use, there is considerable debate about the short- and long-term effectiveness of social advertising as a strategy to tackle obesity. To date, there has been limited published research evidence about: a) how different styles of anti-obesity social advertising strategies may impact upon audience segments in different ways and b) how anti-obesity campaigns impact upon audiences who may not be the intended *targets* of campaigns, but who are *exposed* to campaigns through their routine media use. Wakefield and colleagues [18] argue that social advertising has great potential to disseminate *“well defined behaviorally focused messages to large audiences repeatedly, over time, in an incidental manner, and at a low cost per head”* [pg 1261] [18]. However, they also state that the current design, delivery, content and funding of campaigns, as well as an increasingly competitive media environment, may lead social advertising campaigns to at best fall short and at worst backfire. There is emerging discussion and empirical research about the possible unintended consequences of social advertising [19-21]. For example, empirical research on the unintended consequences of fear-based anti-obesity campaigns, including the stigmatization of obese adults, has concluded that those who have experienced weight stigma have strong negative and defensive reactions to the messages used in these types of advertisements [22-24]. Other researchers argue that there are clear ethical issues with anti-obesity campaigns that overwhelmingly place the responsibility for behavioural change on the individual, while neglecting the role of other important structural or industry causes of obesity [25].

While most evaluations of social advertising campaigns focus on target audiences, we have less knowledge about individuals who may not be the target audience for the campaign but who are exposed to advertisements as part of their routine media use – hereinafter called *incidental audiences*. Wakefield et al. [18] identify that most social advertising strategies throw a broad ‘net’ via mass television and radio campaigns that reaches a wide, but not necessarily well-targeted, audience [18]. While this strategy may ‘catch’ some of the proposed target audience in the net, it is also likely to expose incidental audiences to these messages. They argue that audience reception of these campaigns is relatively passive – for example, being exposed to campaigns in advertisement breaks while watching television shows. What is unclear from current obesity literature is how different types of social advertising strategies may influence the beliefs and behaviours of these incidental audiences.

The present study aimed to explore how parents (a specified target audience) and children (an incidental audience) interpreted and interacted with messages within two major, but very differently framed, anti-obesity social advertising campaigns in Australia: *Measure Up* and *Swap It* (2008 – 2013). The aims of the *Measure Up*/*Swap It* campaigns were to “raise appreciation of why people need to change their lifestyles” and provide “supporting information on ‘what to do’ and ‘how to do it’” [26]. The stated primary target audience of both campaigns was 25 to 50 year old parents, with a secondary audience of 45-60 year olds who may have or be experiencing early signs of chronic illnesses. *Measure Up* used a problem-focused style of campaign to inform individuals about the health risks associated with overweight and obesity. The advertisement showed a man walking along a tape measure getting older and gaining weight, with his young daughter playing alongside him. *Swap It* followed on from *Measure Up*, and was solution focused, using cartoon characters and a fun style of campaigning to provide positive practical advice about how individuals could make small changes to their lifestyles to reduce their weight [27,28].

## Methods

### Recruitment

Two market research companies were used to recruit 150 family ‘groups’ comprised of at least one parent and one child (aged 9-18 years) from metropolitan Melbourne (Victoria) and Adelaide (South Australia). We used a purposive sampling approach to recruit 75 families from each state using Socio-Economic Indicators for Areas Index of Relative Social Disadvantage quotas to broadly represent the population distribution of families across each state. If a parent agreed to be contacted by the research team they were provided with the phone number and email address of the parent. We then provided additional information about the study using a Participant Information Sheet. The parent was given a day to read about the study and discuss this with the family. If they agreed to participate, the research team made an appointment for the interview. Written informed consent was sought from and provided by the parent on behalf of the family group before the commencement of the interview.

Individuals who were not fluent in English were excluded from the study. For ethical reasons, we also excluded children who had a history of eating disorders. Ethics approvals were received from two University Human Research Ethics Committees - the University of South Australia and Monash University - and each family group was provided with a $100 grocery voucher as reimbursement for their participation in the study. Confidentiality was assured and all identifying data (such as names) were removed from the transcripts.

### Data collection

Face-to-face, semi-structured interviews were conducted in family homes between July 2011 and July 2012 by two trained researchers. Interviews lasted 45-120 minutes and were digitally recorded. The recordings were subsequently professionally transcribed. One researcher conducted the interview, while the other researcher took notes relating to the family dynamics during the interviews and any verbal and non-verbal cues of interest.

For the first part of the study, children and parents were separated out of hearing range from each other and were assisted by the interviewers in filling in a short, structured, mixed methods survey (lasting about 20 minutes). The survey firstly included quantitative questions about their socio-demographic characteristics (children: age, gender, weight and height (reported by parents); parents: age, gender, self-reported weight and height, ethnicity, household income and occupations and education levels of each family member). Most parents were able to confidently report weight and health data, but if they were not they were asked to estimate these measurements. Secondly, participants were asked qualitative questions about how often they discussed weight and health, who was involved in their discussions (e.g. friends or family members) and any current or previous attempts to change their weight. For the second part of the study, parents and children were interviewed together and were shown the *Measure Up* and *Swap It* television advertisements on a laptop computer. It is important to note that we did not systematically capture whether or not parents or children had seen these campaigns prior to this study. At first we used broad open-ended exploratory questions to explore participants’ reactions to and interpretations of the advertisements. We followed these up with specific prompts if the topics of interest were not covered spontaneously. Questions were used flexibly, and followed participants’ thoughts and opinions as they emerged in the discussion.

The first set of prompt questions was used to stimulate discussion about perceptions of the visual imagery and target audiences of the campaign. Examples of prompt questions included: What type of person do you think that ad was trying to target? What sorts of things in the advertisement make you think that? Do you think the messages in this advertisement applied to you or someone else?

The second set of prompt questions was used to explore perceptions of the primary and secondary messages within the campaigns. Examples of prompt questions included: What did you think was the main message in that advertisement? Were there any other messages? Did you like anything about those messages? Did you dislike anything about those messages?

The final set of prompt questions encouraged discussion on behaviour change. Examples of prompt questions included: What sorts of techniques or strategies does this advertisement use to encourage individuals to think about their health behaviours? How effective do you think those techniques or strategies would be in changing behaviours? What sort of changes would it encourage people to make? Would this advertisement encourage you to change any of your behaviours – why or why not?

### Data analysis

For the quantitative data, basic descriptive statistics were calculated, and we calculated Body Mass Index (BMI) from the information provided about weight and height, using the internationally recognised BMI cut-offs of 25 for overweight and 30 for obesity in children (2-18 years) and adults (over 18 years) [29].

The interview transcripts were imported into NVivo9 (QSR International, Pty Ltd). We initially used open coding techniques to analyse the qualitative data collected in the interviews. Because of the large amount of data in this study, our analytical process involved a number of steps. The first step involved grouping the data according to the responses to each campaign (*Measure Up* and *Swap It*). Parent data and child data were separated, and we used a linking identifier to match the responses from parents and children within the same family. This step created manageable data sets for us to explore and compare the responses from parents and children to the different campaigns. The second step involved reading each of the groups of responses and identifying codes represented in the data. This iterative process involved reading each of the individual responses and clustering similar responses together. We discussed and reached consensus on a preliminary set of codes that represented each of the key areas of interest – perceptions of visual imagery and target audiences, primary and secondary messages and impact on behaviour. These codes were then used to further group and compare the data within and between parent and child groups. The final step was to use a constant comparative analysis to explore and organise the patterns that emerged within the data, [30,31] and then to developed a broader set of themes (and subthemes) of analysis (presented in this paper) [32]. In particular, we explored the data for links between the presentation of information within the two advertisements (for example the visual imagery, and verbal narratives) and how participants interpreted this information. At this stage, we returned to the literature to help us to explain our findings.

## Results

### Description of the sample

A total of 159 parents and 184 children participated in the study (across 150 family groups). The majority of parents identified as Australian or European (96%) and reported that they had completed high school (77%). They were mostly female (82%) and married or in de facto relationships (82%), with a mean age of 44.7 years (range 27 to 63 years) and mean self-reported Body Mass Index of 28.4 kg/m^2^ (range 18.6-57.2). There were equal numbers of boys and girls in the study, with a mean age of 13.5 years (range 9-18 years), and a mean BMI as reported by parents of 20.0 kg/m^2^ (range 14.2 to 37.1). More than half (57%) of the children exceeded the Australian Government guidelines relating to screen time (< 2 hours per day), and 83% reported meeting the guidelines relating to physical activity (≥ 1 hour per day). Participants’ self-reported weight status, education and household composition were broadly similar to the general Australian population [33].

The following section presents the qualitative findings of the study. We use the term ‘a few’ to refer to less than a quarter of participants; ‘some’ to refer to 25-50% of participants; ‘many’ to refer to 50-75% of participants; and ‘most’ to refer to over 75% of participants.

### Impacts of visual imagery: personal resonance and personal responsibility

Each advertisement had a very different visual imagery. These appealed to, engaged and influenced the parents and children in our sample in different ways. Most parents believed that the advertisement was designed to target all adults (regardless of weight), and particularly parents, as was the intention of the campaign. However, the issue of the weight gain of the actor as he walked down the tape measure towards the camera as an aspect of the visual image was rarely commented on by the adults. In contrast, children appeared more sensitive to the visualisation of the adult’s weight gain, with some commenting that the depiction of the overweight man in the advertisement suggested that it was designed to only target individuals who were already overweight or obese.

*Personal resonance* was a theme that arose when the visual elements of the *Measure Up* advertisement were discussed. The visual imagery used in the *Measure Up* advertisement of an adult male getting older and putting on weight with a female child playing alongside him had resonance with the parents. Most parents identified the advertisement as personally applicable – they felt they were the “right age” or “same stage of life” as the actor in the commercial. Similarly, younger children – and particularly girls around the age of the daughter in the advertisement - believed that the messages in the advertisement applied to them and their families. There were a few non-white Australian parents who did not feel engaged with the visual imagery used within *Measure Up*, in particular the use of white-Australian actors. Some of the child participants thought the message of the advertisement was to warn children of the dangers of becoming ‘fat’ as they got older. While this interpretation made some of these children feel worried about future weight gain, others believed that it this was a good way of illustrating an important message about obesity prevention.

*Personal responsibility* about weight gain and its impacts was raised by some children. Some older children stated that they liked the use of a child within the advertisement, believing that this emphasised that parents’ decisions about their lifestyles and their weight could have an impact on the family as well as the individual. A few commented that the use of a little girl meant that *Measure Up* was telling children to encourage their parents to lose weight:

Some of us will turn out like that, but some of us won’t. But if our parents [are overweight] we can tell them no, that you should lose some weight so then you can play with us and you have more energy.

–Female, 10 years, BMI 14, Normal Weight.

*Personal resonance* again rose as a theme in the responses about *Swap It*. The animated characters led parents to believe the campaign was primarily targeted towards children and families, and about believed that the visual representations of parents interacting with their children could be personally applied to their own lives. However, they responded to the use of animation in the advertisement in different ways. Most parents stated that the use of a cartoon family would appeal to a broad group of individuals with a range of attitudes and behaviours, as it did not involve the depiction of any particular ethnic group. However, a few parents found the use of animation childish and condescending, and thought that it trivialised important and serious message about obesity prevention. Children also believed that *Swap It* had been designed to target children or families. The use of animation, special effects, balloon characters, and colour in the advertisements was noted by about a quarter of the children, all of whom believed the use of these visual aspects made the advertisements eye-catching, appealing and engaging for younger audiences. Children also provided other comments on the purposive use of animation. A few children stated that the use of animated balloon characters meant that the advertisement would be less likely to make obese individuals feel “bad” about themselves and one child commented that *Swap It* was an advertisement that “everyone could take something out of”. Another younger child commented that the use of balloon characters was a clever decision by the advertising agency because it was much easier to “let a little bit of air out of the balloons” than to get a real family to lose weight.

### Interpretation of messages about the risks of and solutions for overweight

When discussing the messages being communicated, parents and children interpreted very different messages from the two different advertisements. The content and delivery style of the verbal messages (the serious risk focused message in *Measure Up* compared to the upbeat, fun practical message in *Swap It*) appeared to influence how different audiences (parents and children) interpreted the information that was presented. Most parents felt that the messages within *Measure Up* were realistic, believable and trustworthy. In relation to the primary intended message/s from *Measure Up*, some parents perceived it as the need for people to measure their waists to assess their health risks, with a small number of children also stating this as the main message. Personal responsibility for weight gain was reported by some parents as a clear message of the *Measure Up* advertisement – individuals need to take responsibility for their diet and physical activity to reduce the long-term health risks associated with obesity. About a quarter of children reported that the key message in *Measure Up* was to “lose weight”, with another quarter perceiving that the key message was to avoid weight gain. Children were highly aware of the personal responsibility framing of the advertisement, stating that it was designed to tell individuals to not “make excuses” for being overweight or obese, or that that it was “up to you” to be healthy.

While some parents commented that the serious and confronting style of *Measure Up* would have “cut through” with audiences, others had very strong negative reactions to the messages and delivery style used in the advertisement. For example, some overweight or obese parents had strong defensive reactions, commenting that the primary message about ‘measurement’ was disempowering, and believed the campaign provided little practical advice about how to lose weight or maintain a healthy weight. Some parents also distrusted the campaign message, stating that the advertisement promoted a crude measure of an individual’s health and wellbeing and that it stigmatised individuals who were obese. For example, one mother stated that *Measure Up* promoted a “panic” mentality associated with weight and health:

I don’t think putting centimetres is appropriate because it depends on your height, your condition, whatever. So saying 82 around the waist for a female; what if she’s six foot and she can carry that weight? Do you know what I mean? Someone’s going to go into a panic, I’m fat, I’m fat, I’m fat. There is enough stigma around about this now.

–Female, 42 years, BMI 25.7, overweight.

Younger children in particular interpreted the *Measure Up* advertisement as having strong moral messages about weight and health - for example, that fatness is “bad” and that weight loss is “good” and “healthy”. About a third of children had a strong negative reaction to the key messages within the ad, stating that the campaign was “scary”, “sad”, “negative”, “depressing” or “defeatist”. Some children commented that they particularly disliked that *Measure Up* did not have a “positive ending”, with a few older children mentioning that the advertisement would make overweight and obese people feel more “guilty”, “ashamed”, “self-conscious” and “bad”:

I don’t know if ads should guilt trip people by saying ‘If you don’t lose weight and you die that you’re going to leave your family behind’.

–Male, 14 years, BMI 21.2, normal weight.

Parents who interpreted *Measure Up* as advising people that weight gain was subtle, gradual and capable of creeping up on people were more likely to respond to the campaign positively, regardless of their weight status. These parents felt that the advertisement gave a clear message for people to “check” or “monitor” their weight as an important indicator of health and wellbeing:

As you go through life, you may not be concentrating or you may not be aware of how you’re gradually putting on weight, or gradually becoming less healthy. So it’s more a check of where are you now, are you turning in to this guy, that kind of thing.

–Male, 45 years, BMI 26.53, overweight.

Like *Measure Up*, some parents perceived that *Swap It* had a strong message about personal responsibility. They felt that it was designed to provide ‘prompts’ to encourage families to think about how they could improve their food and physical activity choices. These parents used these prompts to think about how they could translate the messages in the campaign to their own lives. About a third of parents stated that they liked the positive, friendly, solution-based, encouraging, non-threatening and non-stigmatising obesity prevention message. Just under half of the children commented that they liked *Swap It*. The practical suggestions provided in the advertisements had resonance with both parents and children. Most children interpreted that the specific primary message of *Swap It* - to swap unhealthy behaviours for alternative, but comparable, healthier behaviours. Most children liked that the advertisement gave clear examples for people to act upon, and they were able to recall the examples given, including swapping a big meal portion for a smaller meal portion, swapping inside for outside activities, swapping non-active transport for active transport and swapping unhealthy food for healthier food. Other children liked the broad, positive message that the advertisement gave:

There are opportunities to change your lifestyle. There’s always another way so you can have the healthier option. [Swap It] gives the opportunities of what you can do for your own life to create a better health and lifestyle.

–Female, 17 years, BMI 23.1, normal weight.

Some parents perceived that the messages within *Swap It* promoted “realistic” and “doable” behaviours that could be accomplished by a wide range of different types of individuals and families, regardless of their age, gender, weight status, family structure, or financial circumstances:

It’s a positive message and it gives actual suggestions about what people can do - you know, ride a bike rather than drive the car - and gives a few ideas that people might want to try out for themselves.

–Male, 53 years, BMI 22.3. normal weight.

Some children also liked the suggestion that making small changes could snowball into larger ones, and perceived that these changes could be made without drastically impacting on a person’s life:

I think the message is that if you make the little changes, then they snowball into much bigger changes without, you know, any real great change and drastic change in your life, yeah.

–Female, 12 years, BMI 19.5, normal weight.

Most of the parents who were obese noted that this was one of the only anti-obesity advertisements that they had seen that did not rely on fear appeals, shock tactics or telling individuals “what not to do”. However, a few also felt the advertisement was unrealistic and misleading because it made weight loss seem too simple and easy, without taking into account the social, environmental and financial drivers of behaviour.

A different negative concern was raised by some older children, particularly girls, who were worried that the specific message of ‘swapping big for small’ meals could be potentially problematic for adolescents who were already at risk of engaging in dieting and eating disorders. Some of these children felt that it was more important to make the message about eating healthier foods and engaging in fun activities, rather than eating less.

### Messaging tactics and the impact on perceived behavioural change

Parents and children discussed whether or not they felt that *Measure Up* and/or *Swap It* would be effective in encouraging behavioural change either in themselves or in others. Parents’ responses about ‘encouraging behaviour change’ were mixed. Most believed that *Measure Up* was trying to encourage behavioural change, but they were divided about the effectiveness of the campaign in achieving such a goal. For some parents, the advertisement provided a good “first step” as an “alert”, “warning”, or “wakeup call” for individuals to “reassess” their lifestyles. While the advertisement appeared to give the parents a heightened awareness of their health, very few commented that they wanted to act on the messages within the advertisement. For example, the following parent commented:

It didn’t kind of give me that message, ‘Oh my God, I need to get out and go and start walking’. It kind of made me stop and think, ‘Okay, where am I at?’, and, ‘Am I unhealthy; am I not unhealthy?’ But it didn’t make me want me to get up and go out and you know, get running or whatever.

–Female, 43 years, BMI 23.44, normal weight.

The lack of practical suggestions and tools to encourage behavioural change was cited by some parents as a reason why people would not act upon the message, even when encouraged to think about their weight. Parents used their own experiences as examples of not knowing how to change their behaviour after they had seen the advertisement. For example, one mother stated that when she had first seen *Measure Up*, the whole family had measured their waists and were “shocked” to discover that some members of the family were overweight. However, she also commented that despite finding out that some were “in the danger zone”, they did not know what to do next:

When that ad came out I said, “Quick, get your measuring tape, I want to see if it works”. And I think all of us pretty much we were all shocked and we were all going, “What do we do?”

–Female, 40 years, BMI 21.0, normal weight.

Similarly, a few parents argued that the campaign would be unlikely to stimulate behavioural change because it focused only on “weight”, “waistline”, and “weight loss”, without any discussion about other aspects of an individual’s health. Most the children believed that *Measure Up* was trying to stimulate individuals to change their behaviour, but their perceptions related more to moral responsibility being the motivating message. Younger children in particular believed that the confronting style of the advertisement, and particularly the focus on parents’ moral responsibility towards their children, would have a strong impact on behavioural change. For example, some of the children commented that if parents “loved their kids”, or wanted to fulfil their responsibilities as a “good” mum or dad, then they would “want to lose the weight” after seeing the advertisement. One child stated that *Measure Up* would make obese parents realise that they would not want to hurt their children or let them down:

If they really care about their kids, they can deal with it. If they really love their kids, it’s like that’s going to happen to my kids, I want to lose the weight.

–Female, 10 years, BMI 16.88, Normal Weight.

Finally, some parents believed that the use of scare tactics could alienate people from an important health message. For example, some obese parents in the sample said that they had actively ignored or avoided *Measure Up* because they found the message too “depressive”, “threatening”, and “confronting”. The campaign did make two obese mothers very emotional when describing their reactions to the campaigns, with these mothers noting that they felt the campaigns were personally relevant. For example, one of them commented:

It’s a bit scary, but it’s very relevant. I’m just like, ‘Yep, that’s me, that’s me. Yeah, that’s me’. I can relate to it, I get out of breath and fatigued, and it’s just like I know that I look for the easy option. I mean, I won’t even walk through a shopping centre without a trolley or something…

–Female, 47 years, BMI 41.02, obese.

In contrast to *Measure Up*, most parents believed that *Swap It* would be effective in encouraging behavioural change. They reported that the campaign would persuade families to change their behaviours because it focused on small rather than drastic changes. Parents also felt that slowly trying to shift people’s beliefs about food choices and activity was a much more sustainable approach to encouraging lifestyle changes, rather than campaigns that used ‘shock’ tactics that they believed would only stimulate short-term changes. Similarly, most children believed that *Swap It* would encourage families to make changes to their lifestyles. Some children noted that the physical activity cues were relevant to their own lives and reported that they could envisage themselves making the changes suggested in the campaigns:

[It applies to me] *because most of the time on Sundays when all I want is to have is the day off, I just like to watch TV and all that. But since I’ve been seeing this ad now, it makes me want to go outside, shoot some hoops, sometimes get my bike out.*

–Female, 9 years, BMI 20.8, Normal Weight.

Most parents believed that *Swap It* was effective in countering a perception that weight loss was difficult, took a lot of time and energy, and was expensive. Some commented that the message about not having to give up anything would be important for some people. Many parents stated that they could personally see how they could build the messages in the advertisement into practical changes in their own day:

I felt like I could relate to it and it said, “It’s easy”. To me, it’s an easy strategy to do simple things, small changes that might benefit in the long term. And if you don’t lose weight, you have to benefit, you know, having more exercise, eating smaller portions. You have to benefit from that in the long run.

–Female, 34 years, BMI 23.6, normal weight.

However, some obese parents perceived there were barriers to implementing the strategies suggested in the *Swap It* campaign. For example, the following mother felt that while the messages applied and were relevant to her family, it was easier to maintain their existing food consumption and physical activity patterns:

[The ad] *definitely applies to me because I tend to drive everywhere. I don’t walk anywhere. You know what, if I can go to the shop in two seconds in the car, I’ll go instead of walking. For me, being this weight, it’s harder for me to walk. It actually hurts, so the car is just so much easier. And definitely I just notice how big our meals are here. They’re huge. We have way too much. You know, we actually have people come over, the kids’ friends, and they can’t believe the food that they get when they come here because it’s just the way we eat.*

–Female, 48 years, BMI 45.7, obese.

Most children also felt that they were personally unlikely to act upon the messages, despite the ‘likeability’ of the *Swap It* advertisement. This appeared to be largely due to their perception of the overweight status of the characters. They believed that the ‘unhealthy’ behaviours of obese and overweight people in the advertisement were inconsistent with their own lives and, as such, the messages in the advertisement were not personally relevant to them. Examples included that the characters were perceived to be fat while the children perceived themselves to be thin (and these children were according to the BMI measures provided by their parents), and because the characters were “sitting down, eating unhealthy, and are getting fatter”, while they perceived that they were “healthy”, “fit”, and “young”.

## Discussion

This study provides qualitative insights into: a) whether different types of social advertising strategies may lead to unintended or undesirable reactions from different audience segments and b) the impacts of social advertising strategies on incidental audiences. Three key points for discussion emerge from the study findings.

### The impact of social advertising campaigns on incidental audiences

The use of children interacting with parents in both campaigns and the use of animation and a positive friendly message in *Swap It* created an impression with some children and parents that the advertisements were designed to appeal to children and families, rather than just to the primary audience of 25 to 50 year old parents. In some cases, children interpreted they had a role to remind their parents of their moral obligations not to gain weight for the sake of their families, thus placing an onus on the child to undertake a ‘parenting’ role. This is somewhat similar to ‘pester power’ which has been used as an effective tactic by both commercial marketers and tobacco control campaigners. *Measure Up* stimulated some anxiety for a few children who worried about their own future weight gain and the weight of their parents. This supports the findings of other studies that also found that threat campaigns designed to target adults may have negative impacts on a small proportion of children [23]. This suggests that policy makers should debate whether they will take a deontological approach to social advertising, whereby campaigns are rejected outright on the grounds that it is wrong to create anxiety in audiences, or a utilitarian approach, whereby on balance social advertising campaigns are considered to do more good than harm and hence are implemented [19]. These types of questions could be examined and explored both in formative research and campaign evaluation to assist policy makers in their deliberations about the potential positive and negative implications of their social marketing campaigns.

Children’s interactions with *Swap It* raise a number of questions. The findings suggest that anti-obesity advertisements may not be fully using the opportunity to engage children (alongside their parents) in messages about healthy lifestyles because of the focus on weight as the optimal health outcome. Hesketh et al. (2005) found that parents believed that while their children knew which foods were unhealthy, they felt they did not fully understand the consequences of eating unhealthy food and had not “internalised the messages about eating unhealthy foods in the same way they had embraced the messages about the negative health effects of smoking” [pg 22] [34]. Our study also suggests that the focus of *Swap It* on overweight and obesity meant that children found the messages about healthy eating and activity less personally relevant than adults. Further research should investigate whether messages that depict overweight people may create the perception in children that lifestyle factors are only important if individuals are overweight or obese. This research will have important implications for the framing of a range of campaigns which may aim to reach and engage a broader range of audience segments in obesity prevention strategies.

### The threats that social advertising campaigns may pose for some (incidental) audiences

In this study, individuals who were obese appeared to have a strong defensive reaction to *Measure Up*, but also appeared to lack the efficacy to act upon the practical strategies in *Swap It*. For example, some mentioned their size as preventing them from being able to commence a more active lifestyle and others perceived that campaigns made weight loss seem easy. In the development of social advertising for health it is important to remember that obesity is caused by an interaction between environmental and individual factors. In this study some people felt personally disempowered by the advertising messages – ‘I’ve heard the message but what can I do?’ However, it is important to recognise such disempowerment is likely to be caused by a range of individual and structural barriers rather than simply a lack of efficacy per se.

Studies suggest that fear appeals are the most ‘notorious’ for stimulating ‘boomerang’ effects in audiences, where the audience reaction is the opposite to the intended effect of the message [21]. *Measure Up* did appear generally to create awareness but did not seem to generate the desired behavioural change effects, particularly in those who were most at risk who reacted against the message [19]. While *Measure Up* appeared to connect with some parents, others, especially those who were obese, actively challenged and even refuted the information provided in the advertisement. This finding is consistent with other studies that suggest obese adults perceive that anti-obesity campaigns which are based on threat or fear appeals to be disempowering and stigmatising, and these campaigns may therefore lead to strong defensive or avoidance reactions, or encourage individuals to turn to quick-fix solutions for obesity which are unhelpful in the long term [22-24]. Cho and Salmon [21] comment that dissonance (or distress or discomfort) from social advertising campaigns occurs when individuals have a strong desire or motivation for change, but feel that they lack the ability or support to achieve that change [21]. This is not to say that obese individuals can *never* be engaged with social advertising campaigns. However, those creating campaigns could consider how messages about the risks associated with obesity may be clearly backed up with avenues or support mechanisms for behaviour change within the target audience.

One of the key criticisms of *Measure Up* among our participants was that it did not appear to offer any practical resources or strategies to enable individuals to act upon the messages delivered within the campaign. However, the aim of this advertisement was to stimulate awareness of overweight in the target audience, prior to the dissemination of the more action-oriented *Swap It* advertisement. The negative response of some of our participants indicates that long gaps between the scheduling of campaigns within a series should be avoided to ensure that potential solutions are offered as soon as possible after triggering problem recognition.

### The role and impact of personal responsibility frameworks

The parents and children involved in this study recognised that the two campaigns were about individuals taking personal responsibility for their weight status. Some parents criticised the campaigns for implying that lifestyle changes were easy, or for not tackling the broader issues associated with the causes and consequences of obesity. This raises a clear ethical issue about the extent to which social advertising campaigns place responsibility on individuals, rather than investing in the broader suite of initiatives associated with a comprehensive social marketing approach, which includes addressing industry and environment factors. The central role of food in culture and society is complex, and as such requires a range of comprehensive public health actions. Initiatives from many different community and government sectors are needed to influence policy reform, industry marketing tactics, and the broader structural and environmental factors which are recognised as playing major roles in the development of obesity [21,25]. Research suggests that social advertising campaigns are overwhelmed by commercial advertising strategies which give a very positive message about the consumption of unhealthy products [18,20,35]. A new factor in the mix is the new suite of ‘social responsibility campaigns’, in which organisations promoting unhealthy products argue that they are ‘part of the solution’ in tackling the obesity epidemic [36]. These industry based ‘social responsibility’ campaigns should be monitored, prevented and, where possible, counteracted.

This study had four main limitations. First, the study was conducted in a ‘lab’ type environment of forced exposure in family homes. As Hastings et al. (2004) comment, these types of studies provide situations of artificially high attention, which are unlikely to *“capture phenomena that happen in a naturalistic type setting”* (pg 963) [19]. Second, the study did not illicit information about prior exposure to the campaigns and did not engage these families in any long-term follow-up about whether they still held the same beliefs about the campaigns over time as a function of subsequent exposure to the advertisement on television. Third, this sample was predominantly comprised of families who identified as either White Australian or European. Finally, weight/height data were based on parental reporting. While most parents confidently stated weight/height measurements, some parents provided ‘estimates’ and weight/height measurements may thus have been under or over-reported.

## Conclusion

Well-funded, targeted, social marketing campaigns will play an important role in the prevention and management of obesity. The findings of the present study suggest that social advertisements need to be rigorously tested with key target audiences to ensure they produce the intended changes in awareness, attitudes, and behavioural intentions. Most importantly, policy makers should ensure that campaigns are backed up with a comprehensive range of structural and environmental supports to enable different population groups to act upon messages, including those who have strong defensive reactions to campaigns and those who feel that campaigns are irrelevant for their own lives. Comprehensive evaluations of the impact of these campaigns on both target and incidental audiences will provide important information for the development and increased effectiveness of these campaigns in a competitive messaging environment.

## Competing interests

The authors have no competing interests to declare.

## Authors’ contributions

ST: Study Chief Investigator. Contributed to the study design, data collection, data interpretation and the drafting and critical revision of the manuscript. TO: Study Chief Investigator. Contributed to the study design, data interpretation and the critical revision of the manuscript. SP: Contributed to the data interpretation and the drafting and critical revision of the manuscript. HY: Contributed to the data interpretation and the drafting and critical revision of the manuscript. CD: Research assistant. Contributed to the data interpretation and analysis, and the drafting and critical revision of the manuscript. JH: Study Chief Investigator. Involved in the study design and critical revision of the manuscript. All authors read and approved the final manuscript.

## Pre-publication history

The pre-publication history for this paper can be accessed here:

http://www.biomedcentral.com/1471-2458/14/151/prepub
